# Preparing Health Professional Students for Home Visits: Leveraging Technology and Interprofessional Learning

**DOI:** 10.1093/geroni/igaf122.1923

**Published:** 2025-12-31

**Authors:** Bronwyn Keefe, Jordana Muroff, Craig Slater, Karen Jacobs, Megan Nizza

**Affiliations:** Boston University School of Social Work, Boston, Massachusetts, United States; Boston University, Boston, Massachusetts, United States; Boston University, Boston, Massachusetts, United States; Boston University, Boston, Massachusetts, United States; Boston University, Boston, Massachusetts, United States

## Abstract

Home visits are a key part of being a health and social service professional. However, students often do not receive hands-on, interprofessional learning in class to prepare them for home assessments at field placements or after graduation. This leaves home assessment training to agencies, which can lead to a variety of training depth, standardization, and quality. In this interprofessional project between social work and rehabilitation health sciences, we created six innovative online modules and a live session that guided students through AI-simulated scenarios of home assessments for people with disabilities and older adults. 33 students in social work, occupational therapy, physical therapy, nutrition, and speech-language pathology programs participated in the interprofessional home-based assessment training. Students completed a retrospective pre-/post-assessment of their knowledge related to each of the module’s learning objectives. At the end of the live session, students completed the 10-item Student Perceptions of Interprofessional Clinical Education Revised (SPICE-R). Students answered two Likert-type questions: 1) how helpful the training was in learning about the home setting, and 2) how likely they are to pursue future employment in home-based care. Results show that students had statistically significant pre-post gains related to the learning objectives (p < 0.05), developed positive perceptions of interprofessional practice, and felt more prepared to conduct home-based assessments following the training. Students gained valuable, hands-on experience performing home visits in a low-stakes environment, better preparing them for fieldwork and their careers. These findings support the training’s effectiveness in enhancing knowledge about home-based assessment and comfort with home visits.

